# Recurrent Glioblastoma: From Molecular Landscape to New Treatment Perspectives

**DOI:** 10.3390/cancers13010047

**Published:** 2020-12-26

**Authors:** Cristina Birzu, Pim French, Mario Caccese, Giulia Cerretti, Ahmed Idbaih, Vittorina Zagonel, Giuseppe Lombardi

**Affiliations:** 1 Sorbonne Université, Inserm, CNRS, UMR S 1127, Institut du Cerveau, ICM, AP-HP, Hôpitaux Universitaires La Pitié Salpêtrière—Charles Foix, Service de Neurologie 2-Mazarin, F-75013 Paris, France; cristina.birzu-ext@icm.institute.org (C.B.); ahmed.idbaih@aphp.fr (A.I.); 2 Department of Neurology, Erasmus University Medical Center, Doctor Molewaterplein 40, 3015 GD Rotterdam, The Netherlands; p.french@erasmusmc.nl; 3 Department of Oncology, Oncology 1, Veneto Institute of Oncology IOV-IRCCS, via Gattamelata 54, 35128 Padua, Italy; mario.caccese@iov.veneto.it (M.C.); giulia.cerretti@iov.veneto.it (G.C.); vittorina.zagonel@iov.veneto.it (V.Z.)

**Keywords:** glioblastoma, MGMT, hypermutation, targeted therapy, immunotherapy, new treatments

## Abstract

**Simple Summary:**

Glioblastoma is the most common and aggressive malignant primary brain cancer in adults. The prognosis remains poor following standard-of-care treatment with surgery, radiotherapy and chemotherapy, with a median overall survival of about 15 months. Theoretically, all glioblastoma patients relapse. Once tumors progress after first-line therapy, treatment options are limited and management of recurrent glioblastoma remains challenging. In recent years, new treatments have been tested on recurrent glioblastoma patients. These include immunotherapy, antiangiogenic treatment, targeted therapy and combination regimens. Here, we review these treatment approaches and provide an overview on the molecular characteristics of recurrent glioblastoma.

**Abstract:**

Glioblastoma is the most frequent and aggressive form among malignant central nervous system primary tumors in adults. Standard treatment for newly diagnosed glioblastoma consists in maximal safe resection, if feasible, followed by radiochemotherapy and adjuvant chemotherapy with temozolomide; despite this multimodal treatment, virtually all glioblastomas relapse. Once tumors progress after first-line therapy, treatment options are limited and management of recurrent glioblastoma remains challenging. Loco-regional therapy with re-surgery or re-irradiation may be evaluated in selected cases, while traditional systemic therapy with nitrosoureas and temozolomide rechallenge showed limited efficacy. In recent years, new clinical trials using, for example, regorafenib or a combination of tyrosine kinase inhibitors and immunotherapy were performed with promising results. In particular, molecular targeted therapy could show efficacy in selected patients with specific gene mutations. Nonetheless, some molecular characteristics and genetic alterations could change during tumor progression, thus affecting the efficacy of precision medicine. We therefore reviewed the molecular and genomic landscape of recurrent glioblastoma, the strategy for clinical management and the major phase I-III clinical trials analyzing recent drugs and combination regimens in these patients.

## 1. Introduction

Glioblastoma (GBM) is the most aggressive central nervous system (CNS) primary malignancy in adults, with a median age at diagnosis of 65 years [1]. Annual incidence is approximately 3 per 100,000 per year and increases with age and male sex [1,2]. The standard of care in newly diagnosed GBMM includes maximal safe surgical resection, followed by concurrent radiotherapy and temozolomide (TMZ) and six monthly cycles of adjuvant TMZ [3]. Median overall survival (OS) varies between 12–18 months [4,5] and the 5-year survival in GBM is below 7% [1,6]. In adults, younger age and a good performance status (Karnofsky performance score KPS > 70 or WHO score 0) at diagnosis are favorable prognostic factors [1,4]. After first line medical management, GBM virtually always recurs with poorer prognosis (i.e., median PFS of 1.5–6 months and median OS of 2–9 months) [7,8,9]. 

Once tumors progress after first-line therapy, treatment options are limited and the management of recurrent GBM (rGBM) remains a challenge. Loco-regional therapy may be evaluated in selected cases while traditional systemic therapy showed limited efficacy. In recent years, with greater knowledge of the underlying molecular characteristics, a multitude of new drugs and new combination regimens have been tested for efficacy in rGBM patients. 

In this paper, we review the latest molecular discoveries, the clinical management and the major phase I to III clinical trials on recent treatments in rGBM patients

## 2. Molecular Characteristics of rGBM

### 2.1. MGMT Promoter Methylation in rGBM

It was first discovered over two decades ago that *MGMT* promoter methylation is associated with response to alkylating chemotherapy in GBM patients [10]. The predictive role of this biomarker was completed following confirmation in a randomized controlled clinical trial, and further strengthened in two trials in elderly GBM patients [11,12,13]. Perhaps somewhat less well known is the observation that *MGMT* promoter methylation is also prognostic: GBM patients with a methylated *MGMT* promoter have a longer survival, irrespective of treatment with alkylating chemotherapy. 

Several studies have shown that *MGMT* promoter methylation is also prognostic at the time of recurrence in GBM patients. In general, post-progression survival is around 3–4 months longer in patients harbouring *MGMT*-promoter methylated v unmethylated tumors (10.9 v 7.2 months, 8.4 v 6.6 months, 12.5 v 7.9 months and 13.5 v 8.0 months in studies reported by the German Glioma Network, EORTC 1542 (GSAM), the DIRECTOR trial and the EORTC 26101 trial, respectively) [14,15,16,17]. Most of these studies defined *MGMT* promoter methylation using data from the primary tumor. This is possible since *MGMT* promoter methylation is relatively stable. At least three independent studies on paired primary-rGBM samples demonstrated that methylation status is maintained in approximately 70–90% of tumor samples [15,16,18]. Data therefore indicate that patients harbouring *MGMT*-promoter methylated rGBMs have a slightly better post-progression survival.

Evidence for a predictive effect of *MGMT* promoter methylation in response to alkylating chemotherapy in patients with relapsed or rGBM is quite scarce. One study reported improved outcomes in patients with *MGMT*-promoter methylated v. unmethylated tumors treated with fotemustine, where the opposite was observed when tumors were treated with bevacizumab [19]. As bevacizumab has limited clinical efficacy in GBMs, this study suggests that *MGMT*-promoter methylation is predictive of response to alkylating chemotherapy at tumor progression. However, other studies did not observe such differences between treatment and control (LOMUSTINE) arms in methylated v unmethylated tumors [20,21,22]. Establishing this potential predictive role, therefore, remains to be determined but is important to guide treatment decisions at tumor recurrence. 

### 2.2. The Genomic Landscape of rGBMs

To understand what makes rGBMs unique, and thus expose potential treatment targets, one has to compare differences between tumors at diagnosis and at recurrence. For this review, we will only focus on tumors that were also diagnosed as GBMs (IDH-wildtype, if known) at initial diagnosis: lower grade gliomas (IDH-mutant) that evolve into secondary GBMs represent an entirely different tumor entity with unique evolutionary trajectories. Firstly, and perhaps slightly surprising, the number of mutations in known cancer genes does not appear to increase at tumor recurrence, at least for the majority of tumors [16,23,24,25] (though there is an increase in the overall mutational burden [25]). In line with the stability of the number of mutations in driver genes is the observation that many of them (on average ~80%) are retained in the recurrent tumor [16,24,25,26,27]. One study reported preferential gains of mutations in *LTBP4*, *MSH6*, *PRDM2* and *IGF1R* genes [24], though apart from the DNA mismatch repair gene *MSH6*, these have not been confirmed in other large cohort studies. No common larger chromosomal changes have been documented at tumor progression [16], but some individual gains and losses may show within tumor pairs [28]. Despite this apparent similarity in genetic makeup, there is evidence for gain of selective events in the majority (64%) of recurrent tumors and patients harbouring such tumors have worse outcomes [25]. 

Although this relatively large concordance in the genetic makeup between primary and rGBM is true for the majority of tumors, there are some notable exceptions. Firstly, mutation retention is lower in the case of a distant recurrence [26], though distant recurrences are quite rare. Second, despite a generally high mutation retention rate in driver mutations, there are some marked differences between individual genes. For example, mutations in the *TERT* promoter show the highest mutation retention rate (~90%), whereas mutations in the *EGFR* gene is at the other end of the spectrum with a retention rate of approximately 50% [16,25,29]. Of note, there can be ‘driver switches’ where the same gene (such as *EGFR*) is affected in primary and recurrent gliomas, but the mutation differs [16,24]. Hypermutated tumors are the third main exception to the relatively stable genotype ‘rule’. These are detailed in a separate section of this review.

Cataloguing the retention rate is important for clinicians when designing molecular targeted therapy trials. This is because trials at tumor recurrence are usually based on molecular data from the primary tumor (repeat surgeries are not often performed) and potential loss of a mutation should therefore be taken into account. To give an example, when an objective response rate of ~40% is considered positive, the number of patients to be included in a trial is 41 (assuming a power of 80% and a one-sided alpha of 0.025). However, when the genetic change is lost in 20% of samples, the number of patients to achieve similar power is almost doubled (*n* = 80) [16]. 

Similarities between primary and rGBM are also apparent at RNA level, where unsupervised analysis highlighted a significant overlap between primary and rGBM [30]. Expression-based molecular subtypes are also relatively stable during tumor progression [31,32]. Some changes are however noticeable when looking at the expression of individual genes, for example, in stemness-related genes [33,34]. Methylation classes are also stable at progression in ~85% of cases [31]. This contrasts IDH-mutant low-grade gliomas which, at recurrence, often exhibit lower overall DNA methylation levels, an increase in the frequency of poorer prognostic subclasses and worse outcomes for patients at progression [35,36]. 

Despite this similarity between primary and recurrent glioblastomas, there is evidence for considerable intratumoral heterogeneity in both. For example, spatially separated samples taken from the same resection may differ with respect to their genetic makeup [27,37]. Even if most studies on intratumoral heterogeneity have been performed on primary tumor samples it is therefore likely such heterogeneity also exist in recurrent glioblastomas and may affect treatment response [38]. In summary, recurrent gliomas generally retain the genetic and epi-genetic makeup of the primary tumor and, as such, are likely to require similar treatment regimens.

### 2.3. Hypermutated GBMs

A subset of temozolomide-treated GBMs gain inactivating mutations in DNA damage repair genes, such as *MSH6*, *MSH2* and *MLH1,* as first described in 2006 by the Sanger institute [39]. Because of their impaired DNA repair pathways, these tumors fail to correctly repair the damage inflicted by the alkylating agent and as a consequence, acquire an exceptionally large number of mutations (often > 10 mutations per megabase) [40]. Temozolomide-induced hypermutated tumors are characterized by G:C > T:A transitions within a specific genetic context (COSMIC mutational signature 11) [41,42]. Hypermutated tumors may also arise de novo, which occurs in the context of germline mutations in DNA mismatch repair genes [40,43,44]. Such tumors have mutational signatures associated with mismatch repair pathways [40]. Although hypermutation is common in recurrent (IDH-mutant) low grade gliomas, it is quite rare in rGBMs, with frequencies generally reported in the order of less than 10% (6/89 [24]), 14/186 [16], 16/99 [25] and 0/29 [26]). Hypermutation appears to occur more often in MGMT-methylated GBMs (23%) compared to MGMT-unmethylated tumors (5.6%) [40]. 

Despite the large difference in the genetic makeup of hypermutated tumors, it is unclear whether patients with such tumors have a different clinical course. One report suggested a longer survival [24], although other studies noted no survival differences [16,25,45,46] or even a trend towards poorer survival in IDH-wt rGBMs [40]. There is scarce evidence on the efficacy of treatment of hypermutated GBMs. The effect of alkylating chemotherapy seems limited: a retrospective analysis found highly similar survival between hypermutated and non-hypermutated tumors treated with alkylating chemotherapy [25] and preclinical evidence suggested hypermutated tumors are resistant to temozolomide [40]. Because of their increased mutational burden, it has been speculated that hypermutated tumors may be more susceptible to immune checkpoint inhibition. Initial anecdotal evidence supported this notion [44,47], although a later retrospective analysis of gliomas with high mutational burden found no evidence for this, with no increased immune infiltration [40]. However, evidence in larger trials is thus-far lacking and to date, there are no specific treatment options for hypermutated GBMs [48]

## 3. Management of rGBM

### 3.1. Diagnosis of rGBM

The diagnosis of rGBM relies on clinical status and MRI findings, according to Response Assessment in Neuro-Oncology (RANO) criteria and medical history [49]. MRI features of rGBM are heterogeneously described [50]. GBM may recur: (i) at the initial tumor site—most frequently <2 cm from lesion—in about 80% of cases [50] and/or, (ii) distant, with unifocal/multifocal parenchymal lesions or leptomeningeal spread [51]. Surprisingly, among different localizations, cortical GBMs seem more prone to multifocal recurrence [52]. 

The distinction between disease recurrence and treatment-related complications is challenging and needs specific attention. The main treatment-related complications are pseudoprogression (PsP) and radionecrosis [7]. PsP, more common in *MGMT* methylated GBM, is seen in up to 30% of patients treated with standard of care [53,54]. Usually, PsP is characterized by tumor volume increase within 3 months post-chemoradiation therapy, but delayed cases have been reported [5,55]. This phenomenon is also seen after immunotherapies with a longer time frame leading to the development of dedicated assessment tools: iRANO [54,56,57,58]. Radiation necrosis is another complication seen later in GBM patients treated with both radio and chemotherapy [7,55]. It usually appears between 3-12 months after radiotherapy [55]. In both situations, RANO and iRANO criteria suggest: (i) careful selection of reference imaging, (ii) close clinical and radiological follow-up and, (iii) avoidance of premature discontinuation of a potentially efficient treatment in the absence of worsening symptoms [7,54,58]. Multimodal imaging including spectroscopy MR, dynamic susceptibility MR perfusion and nuclear imaging can help reach a final diagnosis [5,7,50]. The importance of multimodal imaging is even more apparent with blood-brain barrier permeability modifiers, such as antiangiogenic drugs [55]. 

Moreover, functional molecular imaging such as positron emission tomography (PET) using amino acid tracers emerged as a promising investigational strategy in the setting of diagnosis, biopsy, resection and response assessment [59]. Histological proof remains the best approach to get molecular features of rGBM for potential molecular targeted therapies. However, a limited number of rGBM patients are eligible for second biopsy or resection due to their frailty. Therefore, in this setting, multimodal approach including PET and MRI appear an interesting alternative [5].

### 3.2. Prognostic Factors in rGBM 

Older age at diagnosis and a decreased performance score (KPS or WHO) at recurrence have been associated with a poor outcome in multiple cohorts of rGBM patients [4,9,60]. In the same line, localization of recurrence (i.e., contact with SVZ and/or ventricle) and ependymal spread on MRI have been linked to a poor outcome [52,61,62]. In contrast, cortical localization, volume of FLAIR hyperintensities on MRI do not significantly impact outcome [4,61,63]. rGBM localization in eloquent areas and tumor volume [60] time to first recurrence [4] and RTOG-RPA class [9] were also proposed as prognostic indicators, but data are conflicting and warrant further investigations. As described previously, the *MGMT* promoter methylation status can represent an important factor correlating with survival in rGBM patients. 

### 3.3. Treatment of rGBM

Less than 50% of rGBM patients are eligible for second surgery (12–48%) [63,64,65]. When feasible, surgical resection is associated with increased OS (i.e., 5–11 months) and preserved neurological status (i.e., >90% of patients) [4,63,64,65,66,67]. In these studies, an age of less than 65 years, a good performance status, radical surgery, tumor location and chemotherapy treatment before recurrence were founded predictors of re-surgery benefits; in the presence of these clinical and surgical parameters, second surgery at the time of GBM recurrence could be considered as a therapeutic strategy in selected patients. However, the observed increased survival should be taken with extreme caution due to a selection bias of prognostically favorable patients for second surgery. The impact of surgery in rGBM was never assessed in a prospective manner, nor compared to medical treatments.

Reirradiation (re-RT) can be a therapeutic option in rGBM. A secondary analysis of the Radiation Therapy Oncology Group (RTOG) 0525 trial demonstrated a modest clinical benefit of re-RT compared to best supportive care alone in rGBM patients (HR 0.74, 95% CI, 0.43–1.28). This survival benefit is amplified when re-RT is combined with systemic therapies (HR 0.44, 95% CI, 0.30–0.63) [68]. A systematic review and a metanalysis of 50 studies support the benefit of re-RT with a PFS6 of 43% (95% CI, 35–50%, I^2^ = 82%) [69]. However, the lack of prospective trials, the heterogeneity of studies for patients and the radiotherapy regimen limit the drawing of robust conclusions in rGBM [69,70]. Re-RT can only be proposed after careful consideration of the risk of radionecrosis [55]. A phase III trial has currently been withdrawn due to funding issues (NCT01830101). Stereotactic radiosurgery has been shown to be associated with a better PFS6 (47%). It has the theoretical advantage of sparing normal tissue but is restricted to small tumors with well-defined borders - a rare condition in rGBM [7,69].

With regard to systemic treatments in rGBM, multiple therapeutic options may be considered: (i) temozolomide rechallenging [71], (ii) lomustine or bevacizumab or both [14], and (iii) tumor-treating fields [72], but most agents proved to be limited or had no efficacy in randomized trial settings (median PFS of 2–3 months and PFS6 rate below 15% [5,7]). Thus, due to a lack of validated standard of care, the National Comprehensive Cancer Network (NCCN) recommends clinical trials as the preferred option for eligible patients [5,70]. 

## 4. Summary of Major Phase III Clinical Trials

All clinical trials dedicated to recGBM required proof of recurrence based on RANO criteria after first-line treatment. Eligibility criteria are very similar for most studies: (i) age > 18 years, (ii) PS (KPS > 60–70), (iii) IDH-wildtype status, (iv) prior chemotherapy delivered at least 4 weeks before initiation of experimental therapy, (v) last radiotherapy session delivered at least 7 weeks before initiation of experimental therapy and (vi) known *MGMT* promoter methylation status [14,73]. The endpoints are median PFS, median OS, PFS6, and OS12 (OS at 12 months) [14,73,74,75]. 

Multiple cytotoxic chemotherapeutic agents were explored in recGBM without clinical advantage in terms of overall survival. Alkylating compounds (i.e., nitrosoureas) were associated with some benefits with a PFS6 of around 20% and a median PFS at 1.5 months [8,14,76]. Interestingly, Lomustine became the control arm for all major clinical trials, although no clinical trial comparing it to a placebo has been conducted [8]. TMZ was also formerly explored in both TMZ-naïve and TMZ-pretreated patients using different dosing regimens: (i) the classical 5-of-28 days, (ii) metronomic TMZ, and (iii) 1 week on/1 week off regimens. The PFS6 varied between 18% to 48% in TMZ-naïve patients and between 8% to 58% in TMZ pretreated patients [7].

A relatively small phase III clinical trial, comparing the combination lomustine plus the standard of care in newly diagnosed *MGMT* methylated GBM, showed an improved median OS up to 48.1 months [77]. The benefit of this association in rGBM is unclear and limited by its toxicity [5,77]. Other chemotherapy regimens were tested either in monotherapy or in association with TMZ without significant benefit [5,7,70]. 

More recently, anti-angiogenic and molecular targeted therapies (MTT) emerged as promising therapeutic strategies. Multiple actionable molecular abnormality-driven multiple signaling pathways were identified in GBM. Worth noting is that, with the exception of anti-angiogenic drugs, none of the MTT explored in phase II trials reached phase III clinical trials. This evaluation is to be considered for regorafenib, a pan-tyrosine kinase inhibitor (TKI) which has proven efficacy in rGBM when compared to lomustine [20]. Since angiogenesis is pivotal in GBM progression, anti-angiogenic drugs targeting the VEGF-VEGFR axis were explored in rGBM: (i) monoclonal antibodies, i.e., bevacizumab, or (ii) small molecules inhibiting tyrosine kinase (TKI), i.e., cediranib and sunitinib. The addition of bevacizumab in a randomized phase III study prolonged PFS up to 4.2 months when added to alkylating agents in rGBM [14]. The benefit was similar in terms of PFS in a previous clinical trial exploring cediranib with lomustine [76]. In both studies, anti-angiogenic drugs failed to improve the OS, and the increase in PFS can be explained by normalization of the tumor vasculature which limits visibility of the tumor on MRI scans [14,76]. Another multi-TKI targeting angiogenesis in rGBM, sunitinib, is currently being investigated in a phase III trial (NCT03025893).

Both active and passive immunotherapies are currently tested in rGBM. The Check-Mate-143 trial evaluating nivolumab, an immune checkpoint inhibitor (ICI), versus bevacizumab in rGBM was negative [56,73]. Corticosteroid use did not impact survival in the bevacizumab arm, while reduced doses were associated with a better outcome in the nivolumab arm (HR, 0.59; 95% CI, 0.36–0.95). Overall, a trend toward a better outcome was seen in MGMT methylated patients without any baseline corticosteroids treated with nivolumab vs. bevacizumab (17.0 vs. 10.1 months; HR, 0.58;95% CI, 0.30–1.11) [73]. Also noticeable is that patient responses, when detected, are more prolonged in nivolumab (11.1 months) vs. the bevacizumab arm (5.3 months) [73]. Active immunotherapy (i.e., peptidic and dendritic cells vaccines) is also explored in rGBM. The peptide vaccines are still in early phase of trials and are discussed in the dedicated subsection. Dendritic cells (DCs) are professional immune cells presenting antigens for T cells. They induce adaptive immunity [78]. Noteworthily, a phase III clinical trial evaluating autologous tumor lysate-pulsed dendritic cell vaccine (DCVax^®^-L, Northwestern Biotherapeutics Inc., Bethesda, Rockville, MD, USA) plus the standard of care showed encouraging results and a satisfactory safety profile in newly diagnosed GBM [79]. In the same line, an ongoing phase III clinical trial investigating autologous dendritic cell vaccines (i.e., ADCTA autologous dendritic cells co-cultured with autologous tumor antigen) suggests specific immune response in rGBM(NCT04277221) [80]

Viral therapy is explored in rGB, either as monotherapy or in association, in phase III studies (see Table 1). 

Oncolytic viruses are either natural or genetically engineered viral strains designed to infect and/or replicate selectively in tumor cells. After an initial phase of direct cytotoxic activity, a second phase of innate and adaptive antitumor immune response follows usually due to released tumor antigens [78]. One phase III clinical trial investigated Toca 511, an intracavitary released retrovirus that delivers a cytosine deaminase cDNA to GBM cells. This provides conversion of 5-fluorocytosine (Toca FC) in 5-fluorouracil (NCT02414165) [81]. This approach failed to improve survival when compared to standard of care [5,82]. Another viral therapy, ofranergene obadenovec (VB-111), a non-replicating adenovirus carrying a Fas-chimera transgene tested with Bevacizumab did not ensure survival advantage when compared to Bevacizumab alone [75,83]. Overall, a limited number of recurrent GBM respond to immunotherapy. Multiple factors are associated with this low response rate: (i) limited immunogenicity and (ii) local immunosuppression. Translational research may help identification of predictive biomarkers [84,85]. Recently conducted major phase III clinical trials are reported in Table 2. 

Cancer stem cells are likely to be pivotal in rGBM and efforts should be made to evaluate whether specifically targeting this tumor cell population prevents tumour recurrence [86]. Specifically targeting this tumor cell population is promising for eradication of the source of recurrence. Using a CLIA-certified and CAP-accredited drug response assay, a phase III clinical trial is currently testing tailored and personalized chemotherapy versus non-guided chemotherapy in rGBM patients (NCT03632135) (see Table 1).

Worth noting is that quality of life assessment, cognitive testing, treatment-related toxicities and symptomatic treatments are issues that also need to be addressed in rGBM patients. To date, a limited number of clinical trials have investigated this field, mostly in newly diagnosed GBM, but the final results were deceiving, thus highlighting the need for further exploration [5].

## 5. Summary of Major Phase II Clinical Trials 

We now describe the major phase II clinical trials analysing treatments in rGBM patients. Experimental agents have been grouped according to their main mechanism of action. All of these studies are summarized in Table 3 and Table 4.

### 5.1. Tyrosine Kinase Inhibitors 

Tyrosine kinase inhibitors (TKIs) have often been evaluated in several studies for rGBM patients [91,119,120,121,122,123,124,125,126,127,128]. Recently, regorafenib, an oral multikinase inhibitor targeting VEGFR1,2,3, TIE 2, PDGFR, FGFR, KIT, RAF-1, RET and BRAF, was evaluated in the REGOMA trial, a randomized phase II study analyzing regorafenib in rGBM patients [20]; the Italian study enrolled 119 rGBM patients reporting a longer overall survival in patients treated with experimental therapy: 7.4 months compared to 5.6 months with lomustine (HR 0.5, 95% CI, 0.33–0.75; *p*= 0.0009). Moreover, the study also demonstrated a statistically-significant improvement of 6-month progression free survival (6m-PFS): 16.9% (95% CI, 8.7–27.5) in the regorafenib arm compared to 8.3% (95% CI, 3.1–17.0) in the lomustine group. The disease control rate (overall response rate plus stable disease) was higher in regorafenib compared to the control group: 44% against 20%, respectively (*p* = 0.0059). Overall, the treatment resulted in a manageable toxicity profile showing grade 3 liver and skin toxicity in 10% of patients, respectively. Based on these results, NCCN 2020 guidelines included regorafenib as a preferred regimen in rGBM patients and the Italian Agency of Medicine (AIFA) approved the use of this treatment for Italian patients. Two subsequent studies demonstrated a higher efficacy of regorafenib in selected patients with specific molecular alterations such as phosphorylated acetyl-CoA carboxylase [129,130]. Dovitinib, an oral FGFR, VEGFR and PDGFRβ inhibitor, was analyzed in another phase II study [87] where dovitinib was tested in two different populations of rGBM patients: anti-angiogenetic naive patients and anti-angiogenetic drug pretreated patients; however, results showed poor efficacy in terms of prolonging PFS (primary endpoint): mPFS was 2.0m (95% CI, 1.3–3.7) for anti-angiogenic naïve patients versus 1.8m (95% CI, 0.9–1.8) for anti-angiogenic pretreated patients (*p* = 0.03). Cabozantinib, a potent multitarget MET and VEGFR2 inhibitor, was tested in two phase II trials in rGBM patients with or without previous anti-angiogenic therapy [88,89]: the authors showed the following results in the group of patients who did not receive prior anti-angiogenic therapy: 6m-PFS was 22.3% and 27.8% for two different dosages (140 mg/day and 100 mg/day, respectively) and the median OS was 7.7 and 10.4 months, respectively. AKT-pathway inhibitors were also evaluated for rGBM patients given the promising preclinical activity shown both in vitro and in vivo [131,132,133]. Perifosine (PRF), an oral alkylphospholipid with an antineoplastic effect inhibiting the AKT pathway and buparlisib, a pan-PI3K (phosphatidylinositol 3-kinase) inhibitor, were tested in two phase II trials [90], demonstrating good tolerability without an improved survival outcome. Alteration of the cyclin-dependent kinase 4–6 (CDK4-6) pathway is common in several types of cancers, including GBM; a phase II trial evaluated palbociclib, an oral inhibitor of CDK4–6, in rGBM patients with RB1 (Retinoblastoma) proficiency in IHC. Despite adequate penetration in tumor tissue, palbociclib did not improve survival in this setting of patients [92]. Imatinib mesylate is a multitargeted tyrosine kinase KIT, Bcr-Abl and PDGFR inhibitor; this drug was evaluated in recurrent glioma patients (including GBM) in two phase II trials, alone or in combination with hydroxyurea: single agent and combination treatment were well tolerated despite limited antitumor activity [93]. Infigratinib is a selective small molecule pan-FGFR kinase inhibitor which was evaluated in a phase II study, presented at the 2019 Society of Neuro-Oncology (SNO) Meeting [94], in recurrent high-grade glioma patients who harboured FGFR1-TACC1 or FGR3-TACC3 fusion, activating mutations in FGFR1,2 or 3, or FGFR1,2,3 or 4 amplification. Infigratinib was shown to obtain disease control in one-third of rGBM patients, with a reversible and manageable toxicity profile. Larotrectinib, an approved FDA selective TRK inhibitor for the treatment of TRK-fusion cancers, showed an impressive response rate and durable disease control both in metastatic brain disease and in primary brain tumors (including GBM). The study [95], presented at the 2019 American Society of Clinical Oncology (ASCO) meeting, analyzed 18 cases with primary brain tumors [six (32%) with GBM, four (21%) with glioma, three (16%) with glioneuronal neoplasm, two (15%) with astrocytoma, three (16%) patients with otherwise unspecified tumors and six patients with brain metastasis. The median prior systemic therapy was 1 (range 0–6). The study reported a DCR of 100% in 14 evaluable patients with a disease control rate ≥ 16 and 24 weeks in 79% and 71% of patients, respectively; the mPFS was 11 months (95% CI, 2.8-NR). Galunisertib, a transforming growth factor (TGF)-β receptor (R)1 kinase inhibitor, which has shown antitumoral activity in association with lomustine in murine models of GBM, was tested in a phase II randomized trial in association with lomustine vs. galunisertib alone vs. lomustine alone in rGBM patients; galunisertib alone or in association with lomustine failed to improve survival. Proto-oncogene tyrosine-protein kinase Src family (SFK) activation has often been found in patients with GBM associated with greater cell motility and invasion [134]; furthermore, SFK signaling would appear to be upregulated in patients with progressive GBM after bevacizumab therapy [135]. Dasatinib, an oral ATP-competitive multitarget kinase inhibitor, is able to inhibit all members of the SRC kinase family and has been shown to block growth of bevacizumab-induced glioma invasion in xenograft models [134]. Galains et al., reported the results of the randomized phase II study analyzing the efficacy of bevacizumab in combination with dasatinib vs. bevacizumab alone in rGBM patients [107]: the addition of dasatinib did not improve 6m-PFS compared to bevacizumab alone (28.9% [95% CI, 19.5%–40.0%] vs. 18.4% [95% CI, 7.7–34.4%]; *p* = 0.22) with no significant difference in mOS between the two arms (7.3 m vs. 7.7 m; HR 0.96 [95% CI, 0.64–1.43]; *p* = 0.83). The epidermal growth factor receptor (EGFR) gene is overexpressed in about 40–60% of GBM and its hyperactivation causes an increase in cell migration, proliferation and invasiveness with a reduction of apoptosis [136,137]. Erlotinib, an EGFR inhibitor, was tested in several studies for rGBM patients but results were not encouraging. In particular, a randomized phase II trial [97] evaluated erlotinib vs. temozolomide or carmustine in 110 rGBM patients. Treatment was well tolerated but the trial failed to meet the primary endpoint of 6m-PFS: 11.4% (95% CI, 4.6–21.5%) in the erlotinib arm vs. 24% in the control arm and mPFS of 1.8m and 2.4m, respectively. Sorafenib, an oral VEGFR-2, Raf, c-KIT, PDGFR and Flt-3 inhibitor, was tested on rGBM patients in association with continuous daily temozolomide (50 mg/m^2^/day) in a single arm phase II trial [98] demonstrating limited antitumor activity. Cediranib is a tyrosine kinase inhibitor of VEGFR2, C-Kit and PDGFR and although prior phase II trials [138] showed an encouraging 6m-PFS rate of 25.8%, the subsequent randomized phase III study (REGAL trial) [76] demonstrated no superiority of cediranib compared to Lomustine (already discussed above–Section 4. Summary of Major Phase III Clinical Trials). 

At the 2019 SNO meeting, Arrilaga-Romany et al. [99] presented the results of the phase II randomized trial in which cediranib was evaluated in association with olaparib, an oral PARP-inhibitor, versus bevacizumab in beva-naive, adult, rGBM patients; 60 patients were enrolled and the combination failed to increase PFS and OS. Lenvatinib, an oral, VEGFR, c-KIT and RET tyrosine kinase inhibitor, was tested among 32 patients with rGBM in a phase II clinical trial; the agent did not meet the primary endpoint of the study, reporting a PFS of only 1.9 months (95% CI, 0.95–2.73) with a 6 m-PFS rate of 8.3 % and overall survival of 4.11 months (95% CI, 3.02–5.88) [100]. At the 2020 European Society of Medical Oncology (ESMO) meeting, Lwin et al. [101] presented the results of the LEAP-005 study: a phase II basket trial including 31 patients with rGBM, treated with the association of lenvatinib plus pembrolizumab. The authors reported interesting results: the ORR and the DCR were 16.1% (95% CI, 5.5–33.7) and 58.1% (95% CI, 39.1–75.5), respectively. 

The BRAF protein is involved in the mitogen-activated protein-kinase (MAPK) signaling pathway (B-Raf/Mek/Erk proteins) and V600E is the most frequent mutation in the BRAF gene described in gliomas, including pediatric (20%) and adult GBM (6%) [139]. Vemurafenib is a tyrosine kinase inhibitor and is a selective oral inhibitor of the oncogenic BRAF V600 kinase; this drug is approved for the treatment of melanoma patients harbouring this mutation. The VE-BASKET trial [109] enrolled 24 patients with BRAF V600-mutant gliomas, including 11 recurrent high-grade gliomas (six GBM and five anaplastic astrocytoma); for these patients, the median PFS was 5.3 months (95% CI, 1.8–12.9), the median OS was 11.9 months (95% CI, 8.3–40.1) and the ORR was 9.1%. Other works suggested that gliomas could be more responsive to the concurrent use of BRAF and MEK inhibitors dabrafenib and trametinib [140]. The ROAR trial (NCT02034110) [110] was a phase II study analysing the efficacy of this combination regimen in subjects with rare BRAF V600E mutated cancers; among these, 39 recurrent high-grade glioma patients were enrolled and treated with trametinib plus dabrafenib: the ORR was 27% (95% CI, 13.8–44.1) and the DCR was 57%. 

### 5.2. Anti-Angiogenic Therapy

#### 5.2.1. Bevacizumab in Combination with Other Drugs

Although, bevacizumab alone did not improve survival in rGBM compared to standard therapy with lomustine, many trials have explored the possibility of adding other drugs to bevacizumab; however, cetuximab (chimeric antibody against EGFR), erlotinib (EGFR tyrosine kinase inhibitor), sorafenib (VEGFR, PDGFR and RAF kinase inhibitor) and tandutinib (FLT3, c-KIT and PDGFR tyrosine kinase inhibitor) when combined with bevacizumab in phase II trials, achieved similar outcomes to bevacizumab alone in terms of PFS and OS [141,142,143]. More recently, bevacizumab was evaluated in association with new generation drugs or devices, with different targets and different mechanisms of action. In a phase II study [102], panobinostat, a histone deacetylase (HDAC) inhibitor, showed poor results when added to bevacizumab. Also in a phase II trial, vorinostat, another HDAC inhibitor, was evaluated in association with bevacizumab vs. bevacizumab alone [103]: both groups showed similar results with a median PFS of 3.7 vs. 3.9m (*p* = 0.94) and a median OS of 7.8 vs. 9.3m (*p* = 0.64) for the combination regimen versus the single drug, respectively. Recently, Fallah et al. [104] presented a phase II clinical trial assessing the safety and efficacy of bevacizumab plus TTFields in 25 rGBM patients: mPFS was 9.9 months (95% CI, 6.7-NA), 6m-PFS was 71% (95% CI, 0.54–0.94); median Overall Survival (mOS) was 9.9 months (95% CI, 7.3-NA) with a 12m-OS of 42% (95% CI, 0.24–0.74). 

#### 5.2.2. Other Anti-Angiogenic Drugs

Several studies have evaluated the use of drugs with anti-angiogenic activity, other than bevacizumab, for the treatment of recurrent GBM. Cilengitide (CIL) is a cyclic arginine-glycine-aspartic acid peptide inhibitor of the integrins avb3 and avb5, essential for endothelial cell migration and adhesion in the neo-angiogenesis process [144]. Several phase I trials [145,146] have shown encouraging results for CIL use in glioma patients and, for this reason, phase II trials were performed to evaluate the efficacy and safety of CIL in the rGBM population. In a phase II study, Reardon et al. [105] analyzed CIL with two different dosages (500 mg or 2000 mg IV, twice weekly) at first GBM recurrence; the authors reported a 6m-PFS rate of 10% (95% CI, 2.8–23.7) and 15% (95% CI, 5.7–29.8) while the median OS was 6.5 (95% CI, 5.2–9.3) and 9.9 months (95% CI, 6.4–15.7), respectively, in the 500 mg/day arm, the 6m-PFS (primary endpoint) was 10% (95% CI, 2.8–23.7) and overall survival was 6.5 months (95% CI, 5.2–9.3); in the 2000mg/day arm, the 6m-PFS was 15% (95% CI, 5.7–29.8) and overall survival was 9.9 months (95% CI, 6.4–15.7). However, the clinical data that emerged from the CENTRIC [147] and CORE [148] trials analyzing CIL in newly diagnosed GBM patients, did not demonstrate benefits in terms of survival outcome. Aflibercept is a decoy protein composed of extracellular domains of VEGF fused to the Fc portion of immunoglobulin (IgG1), able to block VEGF receptor activity; it showed promising results in a preclinical model of glioma [149] but failed to demonstrate efficacy in a Phase II clinical trial, with 25% of patients removed from the study due to toxicity (CNS ischemia and systemic hemorrhage) [106].

### 5.3. PARP Inhibitors 

Poly (ADP-ribose) polymerase (PARP) is a family of proteins involved in certain processes including DNA repair, maintenance of genomic integrity and apoptosis. PARP inhibitors (PARPi) are currently part of the standard treatment for some types of cancer, but not in the neuro-oncology field, although some preclinical and clinical studies are improving our knowledge on their potential use in brain tumors. For recurrent/relapsed GBM patients, several trials evaluated the possibility of adding PARPi to the standard treatment [150]. Veliparib, an oral PARP1 and PARP2 inhibitor, was tested in a phase I/II clinical trial in combination with temozolomide in recurrent TMZ-resistant GBM patients. The analysis was performed on two different cohorts: bevacizumab refractory (74 patients) and bevacizumab naïve (151 patients). Grade 3/4 myelotoxicity was observed in 20% of treated patients with a median PFS of 2 months (95% CI, 1.9–2.1) for both arms and 6m-PFS of 4.4% and 17% for bevacizumab refractory and bevacizumab naïve, respectively [151]. Another phase I trial evaluated Olaparib, an oral PARP1 and PARP2 inhibitor, in combination with temozolomide in rGBM patients [152]. Grade 3/4 myelosuppression was observed in 20% overall and the median PFS was about 2 months

### 5.4. Depatux-M (ABT-414)

Depatux-M, also called depatuxizumab mafodotin or ABT-414, is an antibody-drug conjugate targeting activated EGFR, conjugated to the toxin monomethylauristatin-F, an anti-microtubule polymerizing agent. The INTELLANCE-2/EORTC 1410 study [108] was a randomized phase II study analyzing this drug alone or in association with temozolomide versus the standard treatment of Lomustine or temozolomide in recurrent EGFR-amplified GBM patients. The study was negative in the primary efficacy analysis, with a median follow-up of 15.0 months; however, the combination arm showed a trend for longer survival compared to standard treatment (HR = 0.71; 95% CI, 0.50–1.02; *p* = 0.062). In a subsequent long-term analysis with a median follow-up of 28.7 months, the difference in overall survival between the two arms was statistically significant with a HR = 0.66 (95% CI, 0.47–0.93); this benefit was more evident in patients relapsing more than 16 weeks after the start of the last temozolomide cycle. The most important grade 3-4 adverse event was a corneal epitheliopathy occurring in 25–30% of patients. 

Recently at ASCO 2020, data were presented on the results of an Italian prospective and observational study [153] analyzing the benefits of the “off-label” use of combination treatment in rGBM patients; this study enrolled 36 patients and although 21 of them (58%) received the experimental treatment beyond second-line, results were very interesting reporting a median overall survival of 8.04 months (95% CI, 5.3–10.7) and a 12-month OS rate of 37%. 

### 5.5. Immunotherapy

A summary of the major clinical trials analysing immunotherapy in rGBM patients is reported in Table 4.

#### 5.5.1. Vaccines 

ERC1671, an allogenic/autologous therapeutic GBM vaccine, composed of inactivated tumor cells mixed with tumor cell lysates derived from the patients and three GBM donors, was evaluated in a randomized phase II study for recurrent bevacizumab-naive patients in association with granulocyte-macrophage colony-stimulating factor (GM-CSF) and cyclophosphamide plus bevacizumab vs. placebo plus bevacizumab. The median OS for patients treated with ERC1671 plus bevacizumab was 12 months vs. 7.5 months in the placebo group [111]. 

Rindopepimut is a vaccine against the GBM-specific EGFR driver mutation, EGFRvIII [154]. It was evaluated in a randomized phase II trial [112], in addition with bevacizumab, in recurrent EGFRvIII-positive GBM patients vs. a control injection of keyhole limpet hemocyanin with bevacizumab. Thirty-six patients were enrolled in the experimental arm and 37 patients in the control arm. 6m-PFS, the primary endpoint, was 28% for rindopepimut compared with 16% for control (*p* = 0.12 one-sided); secondary exploratory endpoints showed a statistically significant advantage of rindopepimut in terms of survival (HR 0.53; 95% CI, 0.32–0.88 *p* = 0.01); however, in the randomized phase III ACT IV study, rindopepimut plus temozolomide failed to improve survival in newly diagnosed GBM patients [57]. In another study, Bloch et al. [113] showed the results from a phase II trial in which the heat shock protein vaccine, HSPPC-96, was tested in recurrent and resectable GBM patients alone or in combination with bevacizumab compared to bevacizumab alone. The study failed to demostrate a survival benefit with HSPPC-96.

#### 5.5.2. Immune Checkpoint Inhibitors

Immune checkpoint inhibition with monoclonal antibodies has shown impressive results in the treatment of several types of cancer [155,156,157,158,159,160,161,162,163,164,165,166,167,168,169,170]. As described above in relation to the Checkmate-143 trial, this approach does not seem to be encouraging in rGBM patients. Nivolumab was also tested as a “neoadjuvant” treatment in a phase II clinical trial [114] where 27 rGBM patients received a single dose of nivolumab two weeks before surgery and then every two weeks thereafter until disease progression: the median PFS was 4.1m (95% CI, 2.8–5.5) and the median OS was 7.3 m (95% CI, 5.4–7.9). Pembrolizumab, a humanized monoclonal IgG4 anti PD-1 antibody, was tested in a phase II trial presented at the ASCO 2018 meeting, as monotherapy or in combination with bevacizumab in recurrent bevacizumab-naive GBM patients at first or second recurrence, regardless of PD-L1 expression [115]. Results were comparable to historical data on bevacizumab monotherapy; indeed, in the combination arm, the 6 month-PFS rate was 26.0% (95% CI, 16.3–41.5) while for pembrolizumab alone it was 6.7% (95% CI, 1.8–25–4); the median OS was 8.8ms in the combination arm (95% CI, 7.7–14.2) and 10.3ms with pembrolizumab alone (95% CI, 8.5–12–5). Moreover, as reported in a recent paper [171], pembrolizumab showed no benefit in a subgroup of recurrent high-grade gliomas with immunohistochemical loss of mismatch repair protein expression, although most of them reported a high tumor mutational burden. Pembrolizumab was also evaluated as a neoadjuvant treatment: the Ivy Foundation Early Phase Clinical Trials Consortium conducted a randomized clinical trial to evaluate the immune response and survival obtained from neoadjuvant and/or adjuvant therapy with pembrolizumab in 35 patients with surgically resectable rGBM [116]. Patients in the neoadjuvant arm reported a significant increase in OS compared to the adjuvant arm, with a median OS of 7.5 ms in the adjuvant-only arm and 13.7 ms in the neoadjuvant arm (HR: 0.39; 95% CI, 0.17–0.94, *p* = 0.04). Median PFS was 2.4 ms in the adjuvant-only group and 3.3 ms in the neoadjuvant arm (HR: 0.43; 95% CI, 0.20–0.90; *p* = 0.03). Molecular analyses showed that neoadjuvant PD-1 blockade can induce the activation of tumor infiltrating lymphocytes with a subsequent increase in interferon response in the tumor microenvironment. 

Durvalumab is a monoclonal antibody against PD-L1 and was tested with or without bevacizumab in a multi-cohort phase II trial in newly diagnosed and rGBM patients. In the recurrent patients treated with durvalumab alone [117], the 6m-PFS rate was 20.0% (90% CI, 9.7–33.0) and the median PFS was 13.9 weeks (95% CI, 8.1–24.0); partial response in 4 patients (13.3%) and stable disease in 14 patients (46.7%) was reported, with a disease control rate (DCR) of 60%. The 6m-OS rate was 59.0% (90% CI, 42.6–72.2). Varlilumab, an anti-CD27 agonist monoclonal antibody showed synergistic activity when combined with immune checkpoint inhibitors in preclinical models [172]; in a subsequent phase II clinical trial [118], 22 patients with bevacizumab-naïve rGBM were treated with varlilumab and nivolumab every 2 weeks; the 12 m-OS rate was 38% (95% CI, 18.6–58.2) in the overall population and 43.6% (95% CI, 18.2–66.7) in the unmethylated cohort (uMGMT), while the Median OS was 9.7 m (95% CI, 6.7–14.8) for all patients and 11.3 m (95% CI, 5.3-NR) for the uMGMT subgroup. 

## 6. Summary of Major Phase I Clinical Trials 

This section discusses available clinical data concerning new agents and new combination regimens evaluated in recent phase I studies. These studies are reported in Table 5.

### 6.1. Small Molecules and Tyrosine Kinase Inhibitors 

The use of small molecules and TKIs has always been an important field of research in neuro-oncology as these drugs usually transfer across the blood brain barrier. The PI3K/AKT/mTOR pathway is often activated in malignant gliomas and could be considered as a possible therapeutic target. Temsirolimus, an mTOR inhibitor, and perifosine, an AKT inhibitor, were evaluated in a phase I study in 35 heavily pretreated patients with recurrent malignant gliomas (17 patients with rGBM) [173]; among the 29 evaluable patients, partial response was reported in one patient (3.4%) and stable disease in 13 patients (45%); mOS was 10.4 m (95% CI, 7.2–16.7) and mPFS was 2.7 m (95% CI, 1.8–9.2). The authors concluded that a combination of mTOR inhibitor temsirolimus dosed at 115 mg weekly and AKT inhibitor perifosine dosed at 100 mg daily (following a loading dose of 600 mg) is tolerable in heavily pretreated patients with recurrence of malignant gliomas, including rGBM. MET inhibition has demonstrated important antitumor activity with the regression of human GBM tumor xenografts [174,175]. Loss of PTEN (phosphatase and tensin homolog, a negative regulator of PI3K) is the most common form of PI3K pathway dysregulation, occurring in around 25–44% of all GBMs [176]; the association of capmatinib (MET inhibitor) with buparlisib (PI3K inhibitor) resulted more effective than the single agent in preclinical and in vivo models. In a multicenter open-label phase Ib/II trial [177], 33 patients with rGBM and homozygous PTEN deletion and PTEN mutation were treated with capmatinib plus buparlisib; treatment-related adverse events (TRAEs) were reported in 84.4% of patients and the most common TRAEs were fatigue, nausea, hypertransaminasemia, depression and hyperglycemia. Focal adhesion kinase (FAK) is a non-receptor tyrosine kinase which is involved in the interaction between the cell membrane and the extracellular matrix. Overexpression of FAK was demonstrated in several types of cancer, including GBM [178,179,180,181]. GSK2256098 is an ATP-competitive, reversible inhibitor of FAK, already tested in a phase I trial for non-CNS cancer [182] showing a good tolerability profile. An open-label, non-randomized phase I trial was performed with rGBM patients [183] to assess safety, tolerability, pharmacokinetics (PK) and clinical activity; thirteen patients were enrolled and treated in three different dose cohorts. TRAEs occurred in >25% of patients, with the most common being diarrhea, fatigue and nausea. Stable disease was observed in three patients. The insulin-like growth factor I receptor (IGF-IR) and its ligands IGF-1 and IGF-2 are also involved in gliomagenesis [184]. AXL1717 is an oral small molecule inhibiting IGF-1R and AKT by reducing their phosphorylation in GBM cells [185]. AXL1717 was evaluated in a phase I clinical trial of recurrent or progressive malignant astrocytomas which previously failed at least one standard therapy [186]; nine patients (eight with rGBM) were treated with an oral suspension of AXL1717 and 4 patients (44%) reported a tumor response. A recent Phase I study evaluated the association of stereotactic radiotherapy (FSRT) with alisertib, a 2nd generation Aurora A kinase (AURKA) inhibitor with anti-neoplastic and radio-sensitization activity, in recurrent high-grade glioma patients [187]. AURKA overexpression has been identified in several types of cancer (including GBM) as a driver of chromosomal instability and consequent aneuploidy; seventeen patients were enrolled (11 GBM) starting from the initial cohort of 20 mg BID to the final cohort of 50 mg BID, all with concurrent FSRT (35 Gy in 3.5 Gy fractions): 6 m-OS for all the cohort was 88.2% and median OS was 11.1 m; 6 m-PFS was 35.3% with an mPFS of 4.9 m. 

### 6.2. Immune Checkpoint Inhibitors 

The Keynote-28 trial was a phase Ib basket study evaluating the safety and efficacy of pembrolizumab in different types of solid tumors, involving 26 patients with rGBM with PD-L1 expression >1% on stromal and tumor cells [188]; patients were treated with 10 mg/kg of pembrolizumab every 2 weeks; ORR (the primary endpoint) was 4% (95% CI, 0.1–20.4) and stable disease was observed in 48% of patients. Another phase I trial explored the possibility of combining pembrolizumab with bevacizumab and hypofractionated stereotactic radiation therapy (HFSRT) in recurrent high-grade glioma patients [189]. Patients were treated with radiotherapy (30 Gy in 5 fractions) combined with bevacizumab (10 mg/kg every 2 weeks) and pembrolizumab (100 mg or 200 mg iv every 3 weeks); the treatment was well tolerated with only grade 1 fatigue and grade 1 proteinuria as the most common adverse events. Treatment was discontinued in only one patient due to grade 3 hypertransaminasemia. Durable objective responses (complete response + partial response ≥6 months) were observed in 53% of patients. The 6 m-OS and 12 m-OS were 94% (16 out of 17 patients) and 64% (seven out of 11 patients), respectively. 

Nivolumab was initially tested on rGBM patients in a phase I safety study (Checkmate-143 which then led to a phase III trial, as discussed previously) alone or in association with ipilimumab (an anti-CTLA4 immune checkpoint inhibitor). Forty patients were enrolled and randomized to receive nivolumab monotherapy at 3 mg/kg every 2 weeks (NIVO3) or nivolumab + ipilimumab 1 mg/kg and 3 mg/kg, respectively, every 3 weeks for four doses followed by nivolumab monotherapy at 3 mg/kg every 2 weeks (NIVO1 + IPI3). Another arm (20 patients) involved nivolumab + ipilimumab at the dosage of 3 mg/kg and 1 mg/kg, respectively, every 3 weeks for four doses followed by nivolumab 3 mg/kg every 2 weeks (NIVO3 + IPI1). The most common treatment-related adverse events (AEs) were fatigue (NIVO3, 30%; NIVO1 + IPI3, 80%; NIVO3 + IPI1, 55%) and diarrhea (10%, 70%, 30%, respectively). Treatment discontinuation due to drug and drug-related adverse events occurred in 10% of patients in the NIVO3 arm, 30% in the NIVO1 + IPI3 arm and 20% of patients in the NIVO3 + IPI1 arm. Partial response was achieved in three patients (NIVO3 = 1 and NIVO3 + IPI1 = 1) and stable disease for ≥12 weeks was shown in eight patients (NIVO3 = 2, NIVO1 + IPI3 = 2 and NIVO3 + IPI1 = 4). Nivolumab monotherapy was better tolerated than combination therapy and the toxicity profile was related to the ipilimumab dosage [56]. 

Atezolizumab, an anti PD-L1 immune checkpoint inhibitor, was tested in a phase I clinical trial [190] among patients with rGBM; sixteen patients with measurable lesions per RANO criteria received atezolizumab 1200 mg every 3 weeks, until progression or unacceptable toxicity: 63% of patients experienced treatment-related adverse events but no grade 4 events were reported; one patient (6%) reported a partial response and three patients (19%) had stable disease. 

Yet, several trials explored the possibility of combining immune checkpoint inhibitors with other molecules in order to enhance the activation of the immune system. LAG3 is an alternative inhibitory receptor target showing promising activity [192]; CD137 is a member of the tumor necrosis factor receptor family increasing antitumor response by altering the tumor microenvironment [193]. The Adult Brain Consortium (ABTC) 1501 trial is a phase I multicenter, multi-arm dose-finding study of anti-LAG (BMS-986016) or anti-CD137 (BMS-663513) alone or in combination with anti-PD1 treatment in patients at first GBM recurrence showing a manageable toxicity profile [191]. Epacadostat is an inhibitor of indoleamine 2,3-dioxygenase-1 (IDO1) that showed antitumor activity in several cancer models, especially when associated with other immunotherapy agents [194]. The ACT15377 trial (NCT03637764) evaluated the combination of isatuximab, a monoclonal antibody binding to the CD38 receptor, with atezolizumab in patients with advanced malignancies, including rGBM; results have not yet been presented to date.

In light of the REGOMA results, an interesting phase I trial analyzing the combination of regorafenib plus nivolumab in a subgroup of rGBM patients is ongoing.

### 6.3. Adoptive Cellular Therapies

Genetically engineered T lymphocytes also known as Chimeric Antigen Receptor T cells (CAR-T cells) elicited increasing interest lately. Targets as EGFRvIII, HER2 and interleukin-13 receptor alpha 2 (IL-13R 2) are explored in rGBM phase I clinical trials (NCT01109095, NCT02209376, NCT01109095) [78]. The highly immunosuppressive tumor microenvironnement and CAR-T cell exhaustion may limit CAR-T efficacy in rGBM supporting combination with ICI as pembrolizumab (NCT03726515) or ipilimumab-nivolumab (NCT04003649) [195]. These associations are currently investigated in phase I clinical trial. Noteworthily, the combination of CAR-T plus ICI needs carefull balancing between the benefits and the toxicities of the therapy [195].

## 7. Conclusions

In recent years, a multitude of novel therapies have shown promising signs of efficacy in rGBM patients. Precision medicine such as the combination of dabrafenib and trametinib in BRAF V600E mutated gliomas, or other tyrosine kinase inhibitors such as regorafenib or NTRK inhibitors, may be used in selected patients. Checkpoint inhibitors such as nivolumab and pembrolizumab failed to improve overall survival, yet their association with tyrosine kinase inhibitors seems more promising. 

However, the responses were observed on a highly selected and very limited patient population. Future work should therefore first focus on better understanding the potential responses and identifying the patient population most likely to benefit. In the longer term, an enhanced understanding of the underlying molecular characteristics and genetic landscape of rGBM is required to identify novel (targeted) therapies and combination regimens. 

## Figures and Tables

**Table 1:** Ongoing major phase III clinical trials in GBM.

NCT Number	Title	Drug Regimen	Expected Enrollment	Start Date
NCT03632135	Standard Chemotherapy vs. Chemotherapy Guided by Cancer Stem Cell Test in rGBM	Diagnostic Test: ChemoID Chemotherapy	300	15-May-18
NCT04277221	ADCTA for Adjuvant Immunotherapy in Standard Treatment of rGBM	ADCTA	118	19-Sep-19
NCT02761070	Bevacizumab Alone Versus Dose-dense Temozolomide Followed by Bevacizumab for rGBM, Phase III	Temozolomide Bevacizumab	210	11-Jul-16
NCT03025893	A Phase II/III Study of High-dose, Intermittent Sunitinib in Patients With rGBM	Sunitinib Lomustine	100	31-Aug-18
NCT02678975	Disulfiram in rGBM	Disulfiram| Dietary Supplement: Copper Alkylating Agents	142	Jan-17
NCT03970447	A Trial to Evaluate Multiple Regimens in Newly Diagnosed and rGBM	Temozolomide Lomustine Regorafenib Radiation	550	30-Jul-19

GBM: Glioblastoma; ADCTA: Autologous Dendritic Cell/Tumor Antigen; NCT: number of ClinicalTrials.gov identifier.

**Table 2:** Recent major phase III studies in rGBM patients.

NCT Number	Drug Regimen	*N*	Median OS (Months)	Median PFS (Months)	PFS6 (%)	ORR (%)	Grade 3/4 Toxicity (%)
NCT02511405	VB-111 + Beva vs. Beva (37)	256	6.8 vs. 7.9	3.4 vs. 3.7	NA	27 vs. 22	67 vs. 40
NCT02017717	Nivolumab vs. Bevacizumab (35)	369	9.8 vs. 10.0	1.5 vs. 3.5	16 vs. 30	8 vs. 23.1	18 vs. 15
NCT01290939	Bevacizumab + Lomustine vs. Lomustine (33)	437	9.1 vs. 8.6	4.2 vs. 1.5	28 vs. 17	41.5 vs. 13.6	64 vs. 38
NCT02414165	TOCA 511/FC vs. SOC (41)	403	11.10 vs. 12.22	NA	45.6 * vs. 51.4 *	NA	30% vs. 25.5%

OS: overall survival; PFS: progression-free survival; PFS6: progression-free survial rate at 6 months; ORR: overall response rate; NA: not available; VB-111: ofranergene obadenovec; TOCA 511; vocimagene amiretrorepvec; FC: flucytosine; SOC: standard of care; * overall survival rate at 12 months (OS-12ms).

**Table 3:** Phase II trials analysing TKI, anti-angiogenic therapy and combination treatments.

Drug	Targets	Patient Profile	Dosage	Efficacy	Radiological Response
Regorafenib [20]	VEGFR1,2,3; Tie2; PDGFR; FGFR; KIT; RAF-1; RET; BRAF	1st Recurrence	Regorafenib 160 mg/day 3 weeks on, 1 week off	mOS: 7.4 m vs. 5.6 m (HR 95% CI, 0.33–0.75; *p* = 0.0009) 6m-PFS: 16.9% vs. 8.3%	DCR: 44% vs. 20% (*p* = 0.0059)
Dovitinib [87]	FGFR; VEGFR; PDGFRβ	1st–4th Recurrence	500 mg 5 days on, 2 days off weekly	mPFS: 2 m (95% CI, 1–3–3.5) in anti-angiogenetic naive vs. 1.8m (95% CI, 0.9–1.8) in anti-angiogenetic pre-treated	NA
Cabozantinib [88]	VEGFR2; MET	1st-3rd Recurrence—Prior anti-angiogenic therapy	100–140 mg/day	mOS: 4.1m (95% CI 1.4–16.7) in 140 mg/day group vs. 4.6m (95% CI, 2.9–5.6) in 100 mg/day group mPFS: 3.3 m in 140 mg/day group vs. 2.3 m in 100 mg/day group	ORR: 8.3% in 140 mg/day group vs. 3.4% in 100 mg/day group
Cabozantinib [89]	VEGFR2; MET	1st-3rd Recurrence—Anti-angiogenic Naive	140 mg/day vs. 100 mg/day	mOS: 7.7 m in 140 mg/day group vs. 10.4 m in 100 mg/day group mPFS: 3.7 m in 140 mg/day group and in 100 mg/day group 6m-PFS: 22.3% in 140 mg/day group and 27.8% in 100 mg/day group	ORR: 17.6% in 140 mg/day group vs. 14.5% in 100 mg/day group
Perifosine [90]	AKT; PI3K	N° Recurrence (Median n° of prior therapies: 5)	100 mg daily	mOS: 3.68 m (95% CI, 2.50–7.79) mPFS: 1.58 m (95% CI, 1.08–1.84) 6m-PFS: 0%	NA
Buparlisib [91]	PI3K	1st–2nd Recurrence—PI3K pathway activated	100 mg/day	mPFS: 1.7m (95% CI, 1.4–1.8) 6m-PFS: 8%	NA
Palbociclib [92]	CDK4–6	1st–3rd Recurrence—RB1 proficiency at IHC	125 mg/day for 21 days q28 days	mPFS: 5.14 weeks (95% CI, 5 days-142 weeks) mOS: 15.4 weeks (95% CI, 2–274 weeks)	NA
Imatinib [93]	KIT; Bcr-Abl; PDGFR	Recurrence	600 mg/day escalated to 800 mg/day 800 mg/day escalated to 1.000 mg/day	mOS: 5.9 m (95% CI, 4.2–7.8) mPFS: 1.8 m (95% CI, 1.7–2.3) 6m-PFS: 16% (95% CI, 8–34)	ORR: 32%
Infigratinib [94]	pan-FGFR	50% with ≥2 prior therapy FGFR1-TACC1 or FGFR3-TACC3 fusions	125 mg/day for 21 days q28day	mOS: 6.7 m (95% CI, 4.2–11.7); mPFS: 1.7 months (95% CI, 1.1–2.8) 6m-PFS: 16% (95% CI, 5.0–32.5%)	ORR: 7.7%
Larotrectinib [95]	TRK	TRK-fusion cancer	100 mg/day	mPFS: 11 m (95% CI, 2.8-NR)	ORR: 36% DCR: 100%
Galunisertib [96]	TGF-β inhibitor	1st Recurrence	Galunisertib + lomustine vs. galunisertib vs. lomustine plus placeboGalunisertib: 150 mg twice a day, 14 days on and 14 days off Lomustine: 100–130 mg/m^2^ every 6 weeks	mPFS: 1.8 m (95% CI, 1.7–1.8) for Galunisertib + Lomustine; 1.8 m (95% CI, 1.6–3.0) for Galunisertib monotherapy; 1.9 m (95% CI, 1.7–1.9) for † Lomustine + Placebo	DCR: 21.5% for Galunisertib + Lomustine; 30.8% for Galunisertib; 30% for Lomustine + Placebo
Erlotinib [97]	EGFR	1st Recurrence	Erlotinib 150–500 mg/day	6m-PFS: 11.4m vs. 24.1m (95% CI, 4.6–21.5)	NA
Sorafenib [98]	VEGFR-2; RAF; KIT; PDGFR	1st–3rd Recurrence	Sorafenib 400 mg twice a day plus temozolomide 50 mg/m^2^/day	mOS: 41.5 weeks (95% CI, 24.1–55.1) mPFS: 6.4 weeks (95% CI, 3.9–11.7) 6m-PFS: 9.4% (95% CI, 2.4, 22.3)	NA
Cediranib [76]	VEGFR-2; C-KIT; PDGFR	1st Recurrence	Cediranib 30 mg/day plus lomustine 110mg/m^2^ every 6 weeks vs. cediranib 20 mg/day vs. lomustine 110 mg/m^2^ every 6 weeks	mOS: 8.0 m for Cediranib 30 mg vs. 9.4m for Cediranib 20 mg plus Lomustine vs. 9.8 m for Lomustine plus placebo (HR 1.43; 95% CI, 0.96–2.13; *p* = 0.10) mPFS: 92 days for Cediranib 30 mg vs. 125 days for Cediranib 20 mg plus Lomustine vs. 82 days for Lomustine plus placebo (HR1.05; 95% CI, 0.74–1.50; *p* = 0.90)	NA
Cediranib plus Olaparib [99]	VEGFR-2; C-KIT; PDGFR/PARP inhibitor	1st–2nd Recurrence	Olaparib 200 mg/day plus cediranib 30 mg/day vs. bevacizumab 10 mg/kg IV every 2 weeks	mOS: 247 days for Olaparib plus cediranib vs. 201 days for Bevacizumab (HR 0.816, 95% CI, 0.43–1.54) 6m-PFS: 14% (95% CI, 4–30) for Olaparib plus Cediranib vs. 30.9% (95% CI, 12.7–51.2) for Bevacizumab	NA
Lenvatinib [100]	VEGFR1-3, FGFR 1–4, C-KIT, RET, PDGFRβ	Recurrence after Bevacizumab treatment	Lenvatinib 24 mg/day q28d	mOS: 4.11 m (95% CI, 3.02–5.88) mPFS:1.9 m (95% CI, 0.95–2.73) 6m-OS: 28% 6m-PFS: 8.3%	NA
Lenvatinib + Pembrolizumab [101]	TKI + anti PD-L1	1st Recurrence	Lenvatinib: 20 mg QD; pembrolizumab: 200 mg Q3W	ORR: 16.1% (95% CI, 5.5–33.7) DCR: 58.1% (95% CI, 39.1–75.5) mPFS: 2.8m (95% CI, 1.6–4.0)	NA
Panobinostat + bevacizumab [102]	Histone deacetylase (HDAC) inhibitor	1st–4thRecurrence	Panobinostat 30 mg 3 times per week, every other week, plus bevacizumab 10 mg/kg every other week	mOS: 9m (95% CI, 6–19) mPFS: 5m (95% CI, 3–9) 6m-PFS:30.4% (95% CI, 12.4–50.7)	DCR:87.5%
Vorinostat+ bevacizumab [103]	Histone deacetylase (HDAC) inhibitor	1st–3rd Recurrence	Vorinostat 400 mg/day on 1–7 and 15–21 days plus bevacizumab 10 mg/kg every 2 weeks vs. bevacizumab 10 mg/kg every 2 weeks	mOS: 7.8m vs. 9.3m, (HR 0.93; 95% CI, 0.5–1.6, *p* = 0.79) mPFS: 3.7 m vs. 3.9 m, (HR 0.63 95% CI, 0.38–1.06, *p* = 0.08)	NA
Bevacizumab + TTFields [104]	Electric fields	1st Recurrence	Bevacizumab 10 mg/kg every 2 weeks) plus TTFields	mOS: 9.9 m (95% CI, 7.3-NR) mPFS: 9.9 m (95% CI, 6.7-NR) 6m-PFS: 71% (95% CI, 0.54–0.94)	NA
Cilengitide [105]	αv3 and αv5 integrin receptors inhibitor	1st Recurrence	Cilengitide 500 mg or 2000 mg twice weekly	500 mg: mOS: 6.5 m (95% CI, 5.2–9.3) 6m-PFS: 10% (95% CI, 2.8–23.7) 2000 mg: mOS: 9.9 m (95% CI, 6.4–15.7) 6m-PFS: 15% (95% CI, 5.7–29.8)	NA
Aflibercept [106]	VEGF trap	1st Recurrence	Aflibercept 4 mg/kg every 2 weeks	mPFS: 12 weeks (95% CI, 8–16)	ORR: 18%
Dasatinib+ bevacizumab [107]	TKI: (SRC kinase family inhibitor)	1st Recurrence	Dasatinib 100 mg/bid plus bevacizumab 10 mg/kg q2w vs. dasatinib 100 mg/bid plus placebo	6m-PFS: 28.9% (95% CI, 19.5–40.0) for Dasatinib plus Bevacizumab vs. 18.4% (95% CI, 7.7–34.4) for Dasatinib plus Placebo	ORR: 18%
Depatux-M+ TMZ [108]	Anti-microtubule	1st Recurrence	Depatux-M 1.25 mg/Kg every 2 weeks plus TMZ 150–200 mg/m^2^ day 1–5 every 4 weeks	mPFS: 2.7 m (95% CI, 2.0–3.8) mOS: 9.6 m (95% CI, 7.4–11.8) 12m-OS: 39.7% (95% CI, 29.4–49.7)	ORR: 10.2% DCR: 44.9%
Vemurafenib [109]	TKI (BRAF V600E)	Any recurrence	Vemurafenib 960 mg twice per day continuously	mPFS: 5.3 m (95% CI, 1.8–12.9) mOS: 11.9 m (95% CI, 8.3–40.1)	ORR: 9.1%
Dabrafenib + Trametinib [110]	TKI (BRAF V600E)	Any recurrence	Dabrafenib 150 mg twice per day plus trametinib 2 mg once dailly	NA	ORR: 27% DCR: 57%

mOS: median overall survival; mPFS: median progression-free survival; 6m-PFS: rate of patients free of disease progression at 6 months; ORR: overall response rate; DCR: disease control rate; TKI: tyrosine kinase inhibitor; TMZ: temozolomide; NA: not available.

**Table 4:** Summary of recent phase II immunotherapy trials.

Drug	Mechanism of Action	Patient Profile	Cohorts /Dose	Efficacy	Radiological Response
ERC1671 [111]	Inactivated tumor cells mixed with tumor cell lysate vaccine	1st Recurrence	ERC1671 plus GM-CSF plus cyclophosphamide plus bevacizumab vs. bevacizumab plus placebo	mOS: 12.1m vs. 7.6m mPFS: 7.3m vs. 5.4	ORR: 75% vs. 25%
Rindopepimut [112]	Tumor specific EGF driver mutation EGFRvIII Vaccine	1st–2nd Recurrence—EGFRvIII positive patients	Rindopepimut plus bevacizumab vs. bevacizumab	6m-PFS: 28% Vs. 16% (*p* = 0.12) mOS: HR 0.53; 95% CI, 0.32–0.88; *p* = 0.01)	ORR: 30% vs. 18% (*p* = 0.38)
HSPPC-96 [113]	Heat Shock Protein Vaccine	1st Recurrence—Histologically confirmed diagnosis of rGBM (II surgery)	HSPPC-96 plus bevacizumab vs. HSPPC-96 vs. bevacizumab	mOS: 7.5 m for HSPPC-96 arm vs. 10. 7m for bevacizumab alone (HR 2.06; 95% CI, 1.18–3.6; *p* = 0.008).	NA
Nivolumab [114]	Anti-PD1	1st Resectable recurrence—Neoadjuvant treatment followed by post-surgical treatment	Nivolumab (3 mg/kg), single dose 2 weeks before surgery followed by nivolumab (3 mg/kg) after surgery every 2 weeks	mOS: 7.3 m (95% CI, 5.4–7.9) mPFS: 4.1 m (95% CI, 2.8–5.5)	NA
Pembrolizumab [115]	Anti-PD1	1st–2nd Recurrence	Pembrolizumab (200 mg) IV q3w plus bevacizumab (10 mg/kg) IV q2w vs. bevacizumab (10 mg/kg) IV q2w	mOS: 8.8 m (95% CI, 7.7–14.2) in the combination arm vs. 10.3 m (95% CI, 8.5–12.5) in the pembrolizumab-only arm 6m-PFS: 26% (95% CI, 16.3–41.5) in the combination arm vs. 6.7% (95% CI, 1.8–25.4) for the pembrolizumab-only arm	NA
Pembrolizumab [116]	Anti-PD1	1st–2nd Resectable recurrence-Neoadjuvant treatment followed by post-surgical treatment	Pembrolizumab (200 mg) IV 14 day (±5) before surgery followed by pembrolizumab (200 mg) IV q3w vs. pembrolizumab (200 mg) IV q3w after surgery	mOS: 13.7 m in the neoadjuvant arm vs. 7.5 m in the adjuvant-only arm (HR 0.39; 95% CI, 0.17–0.94, *p* = 0.04) mPFS: 3.3 m in the neoadjuvant arm vs. 2.4 m in the adjuvant-only arm (HR 0.43; 95% CI, 0.20–0.90, *p* = 0.03)	NA
Durvalumab [117]	Anti-PDL1	Bevacizumab Naïve rGBM	Durvalumab (10 mg/kg) every 2 weeks	6m-OS: 59% (95% CI, 42.6–72.2) mPFS: 13.9 weeks (95% CI, 8.1–24–0) 6m-PFS: 20% (95% CI, 9.7–33.0)	ORR: 13.3%DCR: 60%
Varlilumab [118]	Anti-CD27	1st Recurrence—Bevacizumab Naïve rGBM	Varlilumab (3 mg/kg) IV q3w plus nivolumab (240 mg) IV q3w	mOS: 9.7 m (95% CI, 6.7–14–8) 12m-OS: 38% (95% CI, 18.6–58.2) mOS in the unmethylated population: 11.3 m (95% CI, 5.3-NR) 12m-OS in the unmethylated population: 43.6% (95% CI, 18.2–66–7)	NA

mOS: median overall survival; mPFS: median progression-free survival; 6m-PFS: rate of patients free of disease progression at 6 months; ORR: overall response rate; DCR: disease control rate; GM-CSF: Granulocyte-macrophage colony-stimulating factor; NA: not available.

**Table 5:** Summary of recent phase I trials.

Drug	Mechanism of Action	Patient Profile	Dose /MDT	Efficacy	Radiological Response
Temsirolimus plus Perifosine [173]	mTOR inhibitor and AKT inhibitor	Heavly pre-treated glioma patients	Temsirolimus (115 mg) weekly Perifosine (600 mg load on day 1 followed by 100 mg daily)	mOS: 10.4 m (95% CI, 7.2–16.79 mPFS 2.7 m (95% CI, 1.8–9.2)	DCR: 48%
Capmatinib plus Buparlisib [177]	MET inhibitor and PI3K inhibitor	PTEN deletion and PTEN mutation recurrent GBM patients	Capmatinib tab (300 mg/bid) plus buparlisib (80 mg/day)	NA	NA
GSK2256098 [183]	Focal Adhesion Kinase (FAK) inhibitor	1st–3rd Recurrence	GSK2256098 1000 mg/bid or 750 mg/bid or 500 mg/bid/1000 mg/bid	NA	NA
AXL1717 [186]	IGF-R and AKT inhibitor	1st–3rd Recurrence	AXL1717 400 mg/bid	NA	NA
Alisertib plus FSRT [187]	TKI: (AURORA A kinase inhibitor)	Grade III-IV glioma at 1st–3rd recurrence	Alisertib: 20 mg–30 mg–40 mg–50 mg (BID) FSRT: 35–30 Gy in 3.5–3 Gy fractionsMTD not reached	mOS: 11.2 m 6m-OS: 88.2% mPFS: 4.9 m 6m-PFS: 35.3%	NA
Pembrolizumab [188]	Anti-PD1	RGBM with PD-L1 expression >1%	Pembrolizumab 10 mg/kg	mOS: 14.4 m (95% CI, 10.3-NR) mPFS: 2.8 m (95% CI, 1.9–9.1)	ORR: 4% (95% CI, 0.1–20.4) DCR: 52%
Pembrolizumab plus Bevacizumab plus HFSRT [189]	Anti-PD1	RGBM	Pembrolizumab: 100 mg (two patients) or 200 mg (20 patients) q3w Bevacizumab: 10 mg/kg q3w HFSRT: 30 Gy in five fractions)	6m-OS: 94% 12m-OS: 64%	6m-ORR:53%
Nivolumab plus Ipilimumab [56]	Anti-PD1 and Anti-CTLA4	1st Recurrence	Nivolumab 3 mg/kg q2w (NIVO3) vs. nivolumab 1 mg/kg + ipilimumab 3 mg/kg q3w for four doses, then nivolumab 3 mg/kg q2w (NIVO1 + IPI3). Nivolumab 3 mg/kg + ipilimumab 1 mg/kg q3w for four doses, then nivolumab 3 mg/kg q2w (NIVO3 + IPI1) (investigated in a non-randomized arm)	ORR: −11% (95% CI, 0.3–48.2) in NIVO 3 arm −0% (95% CI, 0–30.8) in NIVO1 + IPI3 arm −10% (95% CI, 1.2–31.7) in NIVO3 + IPI1 arm	NA
Atezolizumab [190]	Anti-PD-L1	1st–2nd Recurrence	Atezolizumab 1200 mg IV q3w	mOS: 4.2 m (95% CI, 1.2–18.8) m-PFS: 1.2 m (95% CI, 0.7–10.7)	ORR: 6% DCR: 25%
BMS-986016 or BMS-663513 plus anti-PD1 [191]	Anti-LAG3 and Anti-CD137	1st Recurrence	-BMS-986016 alone (800 mg) -BMS-663513 alone (8 mg) -BMS-986016 (160 mg) plus anti-PD1 (240 mg) -BMS-663513 (3 mg) plus anti-PD1 (240 mg)	mOS: 4.2 m (95% CI, 1.2–18.8) m-PFS: 1.2 m (95% CI, 0.7–10.7)	ORR: 6% DCR: 25%

mOS: median overall survival; mPFS: median progression-free survival; 6m-PFS: rate of patients free of disease progression at 6 months; ORR: overall response rate; DCR: disease control rate; HFSRT = Hypofractionated Stereotactic Radiotherapy; FSRT = Stereotactic Radiotherapy; NA: not available.
